# Supplementary material for: An immune system for the city: A cluster-randomized trial of a new paradigm for surveillance and control of disease vectors
Source: PLoS Negl Trop Dis. 2026 Jun 22;20(6):e0014464. doi: 10.1371/journal.pntd.0014464 (PMC13340813; doi:10.1371/journal.pntd.0014464)
Supplement: S1 Table — Pair-level records are followed by totals for each arm. (DOCX) [file pntd.0014464.s001.docx]

| **Pair** | **Arm** | **Start date (mm/dd/yyyy)** | **End date (mm/dd/yyyy)** | **Active inspection workdays** | **Additional response workdays** | **Spraying workdays** |
| --- | --- | --- | --- | --- | --- | --- |
| 1 | Control | 12/16/2021 | 06/08/2022 | 36 | 0 | 0 |
| 1 | Intervention | 10/20/2021 | 12/15/2021 | 33 | 11 | 5 |
| 2 | Control | 11/24/2021 | 04/25/2022 | 20 | 0 | 0 |
| 2 | Intervention | 10/20/2021 | 11/23/2021 | 16 | 0 | 0 |
| 3 | Control | 06/29/2022 | 08/09/2022 | 25.5 | 0 | 0 |
| 3 | Intervention | 05/27/2022 | 06/28/2022 | 21.75 | 0 | 0 |
| 4 | Control | 06/06/2022 | 06/28/2022 | 16.5 | 0 | 0 |
| 4 | Intervention | 06/29/2022 | 08/19/2022 | 20 | 0 | 0 |
| 5 | Control | 11/26/2021 | 04/29/2022 | 17 | 0 | 0 |
| 5 | Intervention | 10/20/2021 | 11/25/2021 | 18 | 0 | 0 |
| 6 | Control | 12/09/2021 | 05/25/2022 | 33.5 | 0 | 0 |
| 6 | Intervention | 10/20/2021 | 12/06/2021 | 28 | 0 | 0 |
| 7 | Control | 10/20/2021 | 12/06/2021 | 31 | 0 | 0 |
| 7 | Intervention | 12/07/2021 | 06/03/2022 | 31 | 0 | 0 |
| 8 | Control | 08/19/2022 | 09/20/2022 | 20.75 | 0 | 0 |
| 8 | Intervention | 06/09/2022 | 08/18/2022 | 27.75 | 0 | 0 |
| 9 | Control | 10/20/2021 | 05/16/2022 | 50 | 0 | 0 |
| 9 | Intervention | 05/17/2022 | 10/10/2022 | 56.75 | 18 | 17 |
| 10 | Control | 06/23/2022 | 07/12/2022 | 13 | 0 | 0 |
| 10 | Intervention | 08/05/2022 | 08/31/2022 | 15.5 | 0 | 0 |
| 11 | Control | 10/20/2021 | 11/10/2021 | 15 | 0 | 0 |
| 11 | Intervention | 11/11/2021 | 12/02/2021 | 15 | 0 | 0 |
| 12 | Control | 09/01/2022 | 09/22/2022 | 14.75 | 0 | 0 |
| 12 | Intervention | 08/05/2022 | 08/31/2022 | 16 | 0 | 0 |
| 13 | Control | 09/01/2022 | 10/12/2022 | 24.25 | 0 | 0 |
| 13 | Intervention | 08/05/2022 | 11/03/2022 | 26 | 20 | 0 |
| 14 | Control | 10/21/2021 | 12/14/2021 | 32 | 0 | 0 |
| 14 | Intervention | 12/15/2021 | 05/30/2022 | 30 | 0 | 0 |
| 15 | Control | 10/26/2022 | 01/17/2023 | 39 | 0 | 0 |
| 15 | Intervention | 08/10/2022 | 10/25/2022 | 40.25 | 45 | 9 |
| 16 | Control | 08/22/2022 | 09/28/2022 | 26 | 0 | 0 |
| 16 | Intervention | 09/29/2022 | 11/02/2022 | 23 | 0 | 0 |
| 17 | Control | 10/21/2022 | 11/11/2022 | 14 | 0 | 0 |
| 17 | Intervention | 10/03/2022 | 10/19/2022 | 13 | 12 | 2 |
| 18 | Control | 09/01/2022 | 01/11/2023 | 54.5 | 1 | 8 |
| 18 | Intervention | 01/16/2023 | 05/10/2023 | 51.8 | 48 | 9 |
| 19 | Control | 10/28/2022 | 11/22/2022 | 15 | 0 | 0 |
| 19 | Intervention | 10/03/2022 | 10/27/2022 | 15 | 0 | 0 |
| 20 | Control | 10/11/2022 | 11/24/2022 | 30 | 0 | 0 |
| 20 | Intervention | 11/28/2022 | 01/16/2023 | 33 | 0 | 0 |
| 21 | Control | 11/16/2021 | 09/21/2022 | 36 | 0 | 0 |
| 21 | Intervention | 12/12/2022 | 01/17/2023 | 38.6 | 0 | 0 |
| 22 | Control | 11/23/2021 | 04/27/2023 | 84 | 0 | 0 |
| 22 | Intervention | 01/17/2023 | 05/11/2023 | 67 | 0 | 0 |
| 23 | Control | 12/03/2021 | 07/07/2022 | 47.5 | 0 | 0 |
| 23 | Intervention | 07/08/2022 | 10/19/2022 | 38.25 | 22 | 0 |
| 24 | Control | 10/20/2022 | 01/17/2023 | 43.7 | 0 | 0 |
| 24 | Intervention | 01/18/2023 | 05/05/2023 | 44 | 10 | 2 |
| 25 | Control | 06/10/2022 | 07/14/2022 | 21 | 0 | 0 |
| 25 | Intervention | 04/26/2022 | 06/09/2022 | 21.5 | 0 | 0 |
| 26 | Control | 11/03/2022 | 11/28/2022 | 21 | 0 | 0 |
| 26 | Intervention | 11/29/2022 | 01/18/2023 | 19 | 16 | 4 |
| 27 | Control | 06/22/2022 | 07/27/2022 | 21 | 0 | 0 |
| 27 | Intervention | 05/08/2022 | 06/21/2022 | 20.75 | 0 | 0 |
| 28 | Control | 01/02/2023 | 03/09/2023 | 21 | 0 | 0 |
| 28 | Intervention | 11/07/2022 | 12/23/2022 | 25.7 | 0 | 0 |
| 29 | Control | 11/22/2022 | 12/09/2022 | 17.75 | 0 | 0 |
| 29 | Intervention | 11/14/2022 | 11/29/2022 | 19.4 | 0 | 0 |
| 30 | Control | 12/01/2022 | 12/19/2022 | 14.5 | 0 | 0 |
| 30 | Intervention | 11/14/2022 | 11/21/2022 | 10.2 | 0 | 0 |
| **Total Control** | | | | 855.2 | 1 | 8 |
| **Total Intervention** | | | | 835.2 | 202 | 48 |
