# Supplementary material for: An immune system for the city: A cluster-randomized trial of a new paradigm for surveillance and control of disease vectors
Source: PLoS Negl Trop Dis. 2026 Jun 22;20(6):e0014464. doi: 10.1371/journal.pntd.0014464 (PMC13340813; doi:10.1371/journal.pntd.0014464)
Supplement: S1 Text — (DOCX) [file pntd.0014464.s002.docx]

**Un sistema inmunológico para la ciudad: un ensayo randomizado por conglomerados de un nuevo paradigma para la vigilancia y el control de los vectores de enfermedades**

**Resumen**

Los patógenos transmitidos por vectores siguen apareciendo, causando muertes y afectando a los seres humanos con implacable regularidad. Las estrategias convencionales para el control de los insectos vectores tienen su origen en el ámbito militar; la comunicación es jerárquica, las respuestas son unilaterales y las normas están predeterminadas. Desarrollamos un enfoque alternativo, inspirado en el sistema inmunitario adaptativo, y comparamos ambos enfoques mediante un ensayo aleatorizado por conglomerados en el contexto de una campaña urbana en curso para el control de vectores de la enfermedad de Chagas en Arequipa, Perú. Los clústeres consistían en jurisdicciones geográficas predefinidas de centros de salud y contaban con una media de 2271 hogares. Se asignaron treinta clústeres al grupo inmunológico y treinta al grupo convencional, equilibrados según factores asociados a la probabilidad de infestación por vectores. Tras algunos retrasos, el ensayo comenzó en octubre de 2021. Presentamos aquí los resultados preliminares de un análisis intermedio planificado previamente y programado para marzo de 2023. En el grupo adaptativo se identificaron y confirmaron 23 hogares infestados en 10 focos distintos; en el grupo convencional, solo se identificaron y confirmaron 5 hogares infestados, todos procedían del mismo foco. El enfoque inmunitario fue adaptativo, y se dedicó un mayor esfuerzo tras la confirmación de una infestación (1085,2 días-persona en el grupo inmunitario frente a 864,2 en el grupo convencional; razón de tasas 23/1085,2:5/864,2 = 3,66 [1,49–10,60], valor p = 0,0038). Los enfoques de vigilancia de vectores basados en el sistema inmunitario pueden resultar más eficaces que los convencionales, especialmente en ciudades y otros entornos urbanos complejos.

**Resumen del autor**

Las enfermedades transmitidas por vectores causan muertes y afectan a millones de personas cada año; sin embargo, se evitan muchos más casos de infección gracias a las campañas de control de vectores. Estas campañas se han desarrollado en contextos militares y sus estructuras jerárquicas pueden resultar menos eficaces en entornos civiles. Hemos desarrollado un enfoque alternativo para el control de vectores, inspirado en el sistema inmunitario adaptativo, y hemos demostrado que superó al enfoque convencional en el contexto de una campaña de control de vectores de la enfermedad de Chagas en curso en la ciudad de Arequipa, Perú. Las estrategias de control de vectores deben funcionar a todas las escalas, ser flexibles ante los cambios en objetivos y presupuestos, y aprovechar eficazmente los puntos fuertes de las comunidades que pretenden proteger. El sistema inmunitario ofrece un modelo eficaz para alcanzar estos objetivos.

**Introducción**

Las dificultades a las que se enfrenta una comunidad al intentar controlar un insecto peligroso son, en muchos aspectos, análogas a las que afronta el organismo cuando es atacado por un patógeno. A lo largo de la evolución, los mamíferos han desarrollado una defensa compleja e interrelacionada contra los invasores: un sistema inmunitario perfectamente perfeccionado para detectar y controlar las infecciones. El sistema inmunitario adaptativo se basa en la comunicación entre entidades autónomas para reconocer patógenos y reclutar células efectoras [1]; incluye una combinación de respuestas sistémicas y locales para eliminar los patógenos; y, lo más sorprendente, regula estrictamente estas respuestas y crea memoria para mejorarlas en el futuro [2].

Las estrategias convencionales para el control de vectores, por el contrario, surgieron del ámbito militar, con comunicación jerárquica, respuestas unilaterales y regulación predeterminada [3]. A pesar de su omnipresencia, las campañas de control de arriba hacia abajo rara vez han sido cuestionadas formalmente por enfoques alternativos. Llevamos a cabo un ensayo aleatorizado por conglomerados para evaluar un nuevo paradigma para el control de insectos peligrosos, inspirado en el sistema inmunitario adaptativo, frente a un sistema convencional de arriba hacia abajo, en un programa de control de vectores de la enfermedad de Chagas en curso en la ciudad de Arequipa, Perú. Aquí presentamos los resultados preliminares de un análisis intermedio previamente planificado.

La enfermedad de Chagas, causada por el parásito *Trypanosoma cruzi*, es una de las principales causas infecciosas de morbilidad y mortalidad en las Américas [4,5]. Desde 1991, Triatoma infestans, el principal vector de *T. cruzi* en Sudamérica, ha sido objeto de un programa de eliminación conocido como la Iniciativa del Cono Sur. Esta iniciativa, formalizada a principios de la década de 1990 durante un período de retorno a la democracia en muchos países de la región, mantuvo, no obstante, los enfoques convencionales de control de vectores de arriba hacia abajo que habían demostrado su eficacia bajo regímenes más autoritarios. Según muchos indicadores [6,7], la iniciativa ha sido un éxito rotundo, ya que Chile [8],  Brasil [9],  Uruguay [10] y Paraguay [11] lograron interrumpir la transmisión de T. cruzi por T. infestans. Perú no se unió inicialmente a la Iniciativa del Cono Sur, y los esfuerzos de control en el país se retrasaron durante muchos años [12]. *T. infestans* se limita al sur de Perú, donde infestó Arequipa, una capital regional de más de un millón de habitantes [13].

Inicialmente, con el apoyo de la Agencia Canadiense de Desarrollo Internacional y la asistencia técnica de la Organización Panamericana de la Salud, el Ministerio de Salud del Perú llevó a cabo un tratamiento con insecticidas en más de 80 000 viviendas, lo que evitó una mortalidad y una morbilidad significativas [14,15]. La campaña, al igual que muchas campañas convencionales [16,17], constó de tres fases: la fase de planificación preliminar, que sirvió para definir las áreas geográficas que requerían control [18]; la fase de ataque [19], durante la cual brigadas de técnicos en control de vectores aplicaron insecticidas piretroides en las áreas domésticas y peridomésticas de todos los hogares participantes (dos veces con un intervalo objetivo de seis meses); y la fase de vigilancia posterior a la fumigación, en la que se monitoriza la presencia de insectos en los hogares mediante una combinación de métodos pasivos y activos.

La fase de ataque en Arequipa se prolongó de 2003 a 2018 y se llevó a cabo distrito por distrito. Los estudios serológicos realizados simultáneamente a la fase de ataque revelaron una prevalencia de la infección que oscilaba entre el 7,6 % en un distrito perirrural (La Joya) [20], alrededor del 5 % en distritos periurbanos [13], el 2,3 % en distritos más céntricos (varios distritos) [21] y el 1,4 % en Mariano Melgar [22]. Los distritos entraron en la fase de vigilancia de forma escalonada, seis meses después de completar la aplicación de insecticidas. Inicialmente, las actividades de vigilancia posteriores a la fumigación se limitaron principalmente a la vigilancia pasiva, con búsquedas activas ocasionales de hogares infestados cuando se contaba con personal [23,24]. Se puso a prueba una serie de planes de vigilancia basados en datos en varios distritos de la ciudad [14,25,26]. Estos estudios incluyeron la búsqueda del insecto en más de 8000 hogares basándose en los datos recopilados durante la fase de ataque y en los nuevos datos acumulados durante la fase de vigilancia. Solo se detectaron tres hogares infestados gracias a estas iniciativas.

**Materiales y métodos**

**Diseño de la intervención**

*El enfoque inmune*: Adaptamos aspectos del sistema inmune desde la escala celular hasta la del paisaje, incorporándolos a las actividades en curso de vigilancia vectorial de la enfermedad de Chagas. Nuestra analogía se describe en detalle en la Figura 1. El enfoque inmune constituye el brazo de intervención. *El enfoque convencional* es una versión ampliada de la práctica estándar, que incluye técnicas tradicionales de comunicación y vigilancia pasiva, junto con métodos de vigilancia activa de vanguardia, aunque de carácter vertical. El enfoque convencional se implementó en el ensayo como el brazo de control. A continuación, describimos las principales formas en que ambos enfoques divergen, tanto a nivel ideológico como metodológico.


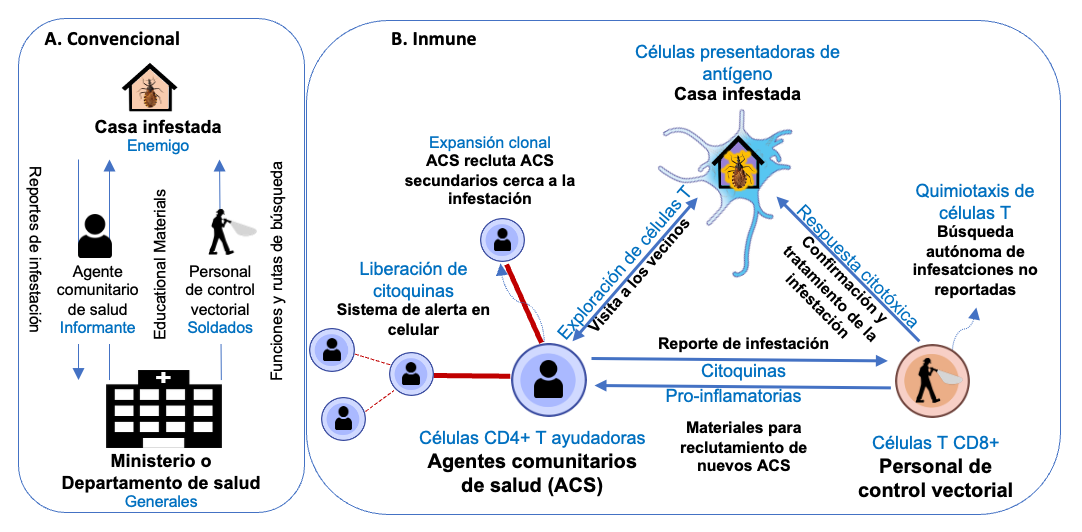


**Figura 1**. Analogías para el control de vectores: a) la analogía convencional, de carácter militar; b) la analogía con el sistema inmune. CHW = agente comunitario de salud. La imagen incluye iconos procedentes de <https://bioart.niaid.nih.gov> y openclipart.org bajo la licencia CC BY 4.0. Concretamente, el icono «Casa» se ha tomado de openclipart.org/74425; el icono «Célula T» de <https://bioart.niaid.nih.gov/bioart/509>; el icono «Avatar sencillo» de openclipart.org/307452; el icono «inspector» de <https://openclipart.org/image/800px/163837>; el icono «Hospital» de openclipart.org/314202; y el icono «célula presentadora de antígenos» de <https://bioart.niaid.nih.gov/bioart/284>. El icono del triatomino fue diseñado por nuestro equipo.

**Reportes o denuncias**

El sistema inmunitario es extraordinariamente igualitario. Cualquier célula de una gran población puede presentar un antígeno y activar el sistema, lo que permite detectar a tiempo un patógeno invasor, antes de que se propague.

En el enfoque convencional, los hogares infestados no se consideran células presentadoras de antígenos que buscan la atención del sistema de vigilancia para enfrentar al enemigo, sino que, más bien, se les considera el "enemigo". Las autoridades de salud pública desconfían de los propietarios y señalan que estos ocultan infestaciones o reportan falsamente solo para obtener gratuitamente el tratamiento con insecticida. La falta de confianza se manifiesta en las normas tanto para la notificación como para la búsqueda (esta última se analiza más adelante). En cuanto a la notificación, si un propietario encuentra un triatomino, está obligado a capturarlo [27] y llevarlo a un centro de salud de atención primaria para su identificación. Este requisito supone una barrera para aquellos propietarios que se muestran reacios o no pueden atrapar insectos [28]. También plantea retos logísticos, ya que el centro de salud debe contar con personal disponible para identificar el insecto o para remitirlo a la persona adecuada de manera oportuna. Además, se necesitan sistemas eficientes de gestión de datos para transmitir la identificación a través de la cadena de mando hasta la oficina central de control de vectores. Pueden producirse retrasos adicionales en la oficina central, donde están las autoridades y los recursos para programar el tratamiento de la vivienda infestada.

En el brazo inmune, eliminamos el requisito de llevar un insecto al puesto de salud para su identificación. En su lugar, aceptamos reportes a través de llamadas telefónicas, redes sociales, mensajes de texto y WhatsApp, mediante un sistema integrado que difundimos con el nombre de AlertaChirimacha’ [29], denominado así por el nombre local del triatomino, chirimacha. Solicitamos, pero no exigimos, fotografías de los insectos. Todos los reportes fueron confirmados por el personal del estudio.

**Búsqueda**

El sistema inmunitario es descentralizado; los linfocitos T guían sus propios movimientos, ayudados por la información que les transmiten las citocinas, de manera muy similar a la forma en que los animales exploran su entorno en busca de alimento [30]. No existe un centro de control; ninguna estructura fisiológica dirige a los linfocitos T. Los paradigmas convencionales de vigilancia de vectores, en contraste, son jerárquicos, con una autoridad central que dirige las actividades del personal de control de vectores. Utilizamos dos aplicaciones, ambas basadas en los mismos modelos de riesgo espacio-temporal [25]. La aplicación de control asignaba a los inspectores los hogares a inspeccionar y proporcionaba alternativas según fuera necesario. La aplicación del enfoque inmune, llamada VectorPoint, ofrecía estimaciones de riesgo, pero permitía a los inspectores utilizar su propio criterio al buscar insectos en sus áreas [14,26]. En ambos grupos no se aplicó un límite estricto de horas de trabajo para la búsqueda, debido a la enorme variación en el tamaño de las viviendas de la zona de estudio.

**Interacción con los propietarios**

Las células T obtienen información de las células somáticas que vigilan, pero no ingresan a ellas. En el sistema convencional, se ordena al personal de control de vectores que entre e inspeccione minuciosamente las viviendas que se les asignan. Este requisito se debe, en parte, a la sospecha de que los propietarios podrían estar ocultando infestaciones que podrían detectarse mediante una inspección exhaustiva. En el grupo inmune no exigimos al personal de control de vectores que entrara en los hogares. En su lugar, se les indicó que visitaran las casas, hablaran con los residentes sobre la enfermedad de Chagas y los triatominos, y proporcionaran materiales que indicaran cómo notificar estos casos a través de AlertaChirimacha. Los inspectores se ofrecían a inspeccionar los hogares si un residente temía que pudiera haber una infestación o si no podía comprobar por sí mismo la presencia de insectos.

**Respuesta**

En el grupo de control de nuestro estudio, se mantuvo la práctica de inspeccionar las viviendas vecinas inmediatas de cada hogar en el que se detectara una infestación por triatominos. En el grupo inmune, el sistema se activó temporalmente tras confirmarse la infestación en un hogar. Se trasladó a técnicos especializados en vectores que trabajaban en zonas cercanas para que ayudaran a controlar el foco de infestación (un radio de 200 m alrededor de la vivienda índice), del mismo modo que las células inmunitarias se desplazan al lugar de la infección. Junto con el técnico original, distribuyeron folletos en todos los hogares y colocaron carteles en las tiendas locales. Además, se publicaron mensajes en Facebook dirigidos a la zona durante 10 días (el alcance de las publicaciones dirigidas de Facebook se define por el número de usuarios de la zona, pero solía ser de alrededor de 1 km) y se incluyó el nombre o los nombres de los barrios afectados para que el mensaje fuera más preciso. Las publicaciones animaban a los residentes a inspeccionar sus hogares en busca de triatominos y a informar de cualquier insecto sospechoso o de sus rastros (heces o exoesqueletos), a través de WhatsApp, mensaje de texto o llamada telefónica. La página de Facebook y las publicaciones eran de acceso público. LDT y CECP supervisaron activamente las publicaciones y respondieron a los comentarios o preguntas. Los comentarios recibidos en las publicaciones se abordaron mediante respuestas públicas, mientras que los informes de posibles infestaciones se gestionaron mediante comunicación directa con los usuarios.

Si se encontraban viviendas infestadas adicionales cerca del foco inicial, el radio se ampliaba alrededor de la nueva infestación y se reclutaban inspectores adicionales según fuera necesario. Se publicaba una entrada de seguimiento en Facebook a los 10 y 30 días de la publicación inicial. Las publicaciones posteriores informaban a los vecinos de que se habían detectado más viviendas positivas y les pedían que permanecieran atentos a la presencia de triatominos y que los reportaran si los encontraban. Una vez que todos los técnicos reclutados para el sitio habían visitado todas las viviendas del área afectada, podían regresar a sus respectivas áreas de trabajo. Publicamos un mensaje de “reducción de la alerta” en Facebook una vez que todas las viviendas infestadas habían sido tratadas y transcurridos 30 días sin infestaciones adicionales. Este mensaje explicaba que las viviendas afectadas habían sido tratadas con insecticida y que los insectos habían sido controlados.

**Desviaciones del protocolo debido a la "inmunosupresión" durante la COVID-19**

El protocolo original asignaba funciones importantes a los agentes comunitarios de salud (ACS). En el brazo convencional, estas personas debían visitar a sus vecinos con regularidad para preguntarles si tenían chirimachas y, en caso afirmativo, informar al centro de salud correspondiente sobre ese hogar. En el brazo inmune, el papel de los agentes comunitarios de salud se inspiró en el de las células T CD4+ ayudadoras. Por lo tanto, se esperaba que visitaran a sus vecinos y, en caso de que alguno informara de una infestación, se comunicaran directamente con el especialista en control de vectores a través de WhatsApp. A continuación, el especialista en control de vectores acudiría al lugar y, si fuera necesario, activaría la respuesta, tal y como se ha descrito anteriormente. Lo más importante era que el agente comunitario de salud  debía “expandirse clonalmente” mediante la capacitación de sus vecinos para que estos, a su vez, sirvieran temporalmente como promotores de salud comunitaria, mientras el sistema inmune se activaba. Estos agentes comunitarios de salud secundarios formarían entonces parte de la memoria del sistema, disponibles para su reactivación si surgiera otra infestación en la zona en el futuro. Durante la pandemia, el sistema de agentes comunitarios de salud colapsó y estas actividades previstas se suspendieron.

**Diseño del estudio**

Dividimos el área de la ciudad bajo vigilancia vectorial en 60 grupos de vigilancia, siguiendo las zonas de influencia existentes del Ministerio de Salud. Con el fin de equilibrar estos grupos (30 en el grupo inmunitario y 30 en el grupo convencional), primero creamos pares utilizando un algoritmo de emparejamiento no bipartito (paquete de R «nbpMatching») [30] y, a continuación, equilibramos la asignación de los miembros de estos pares a los grupos del ensayo basándonos en tres criterios que previamente habíamos identificado como indicadores del riesgo de infestación [24]: el número total de hogares, el número de viviendas infestadas detectadas en actividades de vigilancia previas y los años transcurridos desde la aplicación de insecticida durante la fase de ataque. El tamaño medio de los clústeres en los brazos inmune y convencional fue de 2275,7 y 2266,7 hogares, respectivamente. En el brazo inmune se había tratado una media de 1267 viviendas durante la fase de ataque en cada zona de influencia, y se encontró que una media de 98,5 % de las viviendas estaban infestadas en el momento del tratamiento. En el grupo convencional se trataron una media de 1143,8 viviendas por zona de influencia, y el número medio de hogares infestados fue de 94,6 (Tabla 1). El estudio está registrado en socialscienceregistry.org (ensayo 10985).

El resultado principal del ensayo fue la presencia de insectos en los hogares. Todos los casos de infestación en los hogares fueron confirmados por personal del estudio debidamente entrenado, tanto en el brazo inmune como en el convencional, independientemente del método mediante el cual se detectaran los insectos (vigilancia activa o pasiva). Un resultado secundario fue el número de focos de infestación, considerando como tal a todos los hogares infestados situados a menos de 200 metros entre sí. Comparamos la relación entre el número de infestaciones confirmadas detectadas en cada grupo y los días-persona de búsqueda activa. Aunque no se especificó para el análisis intermedio, también realizamos una regresión de Poisson para datos de recuento emparejados, con un desplazamiento de días-persona en cada conglomerado y un efecto aleatorio entre los pares emparejados.

Tabla 1. Resumen del estudio

| **Componente del estudio** | **Descripción** |
| --- | --- |
| Perido de estudio | Previsto: marzo de 2020-febrero de 2025; análisis intermedio previsto para marzo de 2023. Real: octubre de 2021-marzo de 2023. |
| Diseño de estudio | Ensayo aleatorizado por conglomerados. |
| Tamaño muestral | Se incluyeron sesenta conglomerados, con 30 conglomerados por grupo. El número medio de hogares por conglomerado fue de 2275,7 en el grupo de intervención y de 2266,7 en el grupo convencional. |
| Aleatorización | Los brazos del estudio se equilibraron por número de hogares, tiempo transcurrido desde el tratamiento durante la fase de ataque de la campaña de control y prevalencia de infestación detectada durante la fase de ataque. |
| Clusters | Los clústeres se definieron como las jurisdicciones geográficas de los centros de atención primaria, según el Ministerio de Salud local. |
| Resultados | Resultado primario: número de hogares con presencia confirmada de *Triatoma infestans*, verificada por el personal del estudio. Resultado secundario: número de focos de infestación (hogares infestados situados a menos de 200 m entre sí). |

**Resultados**

En el brazo inmune se detectaron 23 viviendas infestadas: diez en la detección inicial mediante los sistemas ya mencionados y 13 adicionales cuando se activó el sistema en las zonas afectadas. Los diez hogares detectados inicialmente pertenecían a nueve catchments distintos. Cinco fueron reportados inicialmente a través del sistema AlertaChirimacha, ya sea vía Facebook o WhatsApp. Tres se detectaron mediante la búsqueda activa de los especialistas de control vectorial, y dos se reportaron o notificaron a los puestos de salud. En contraste, en el brazo convencional se detectó un total de cinco hogares únicamente en un catchment; una vivienda llevó un insecto al puesto de salud del área, y las cuatro viviendas restantes fueron vecinos inmediatos o colindantes de la vivienda que presentó el reporte original (Fig. 2; Tabla 2).


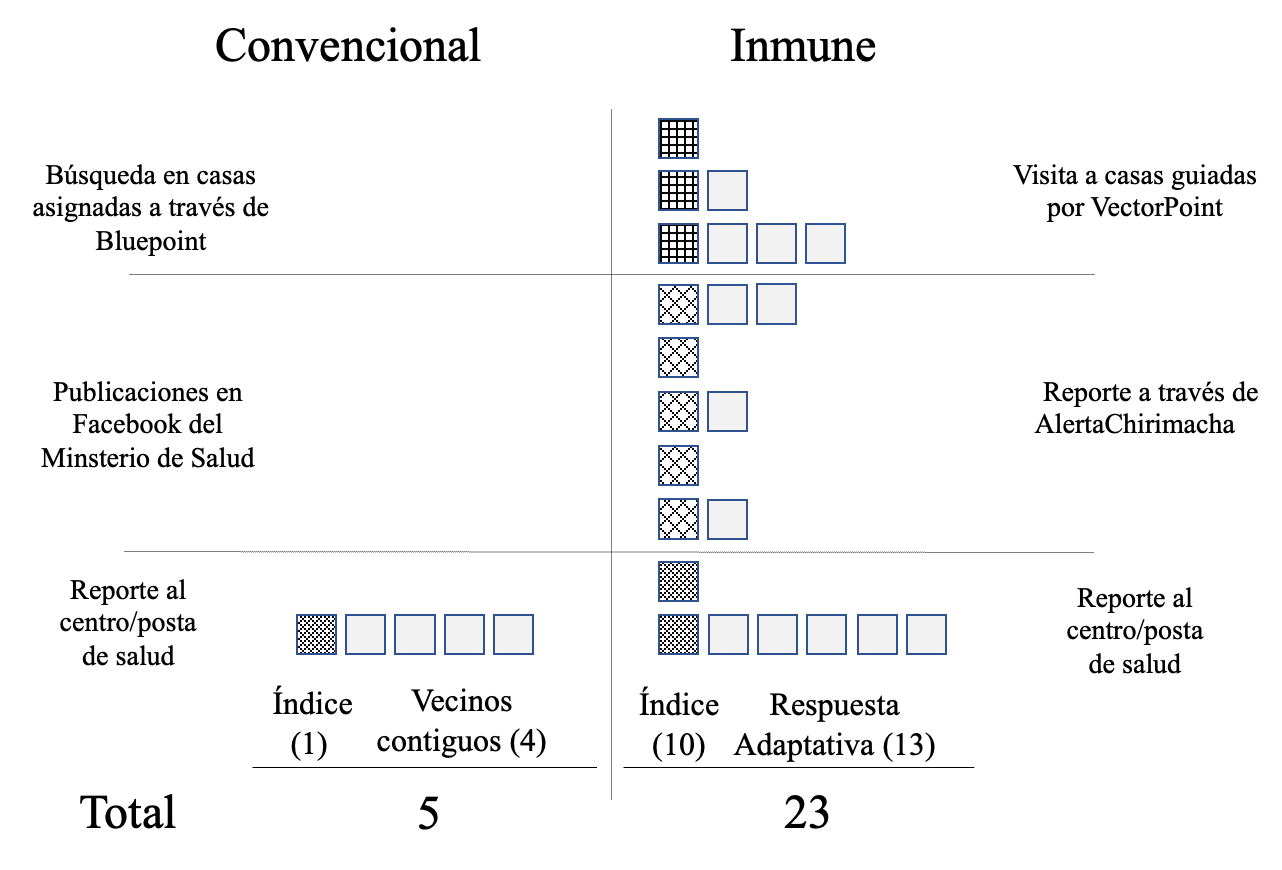


**Figura 2.** Infestaciones detectadas, por forma de detección, bajo los brazos de vigilancia inmune y convencional. Modalidades en inmune: AlertaChirimacha, incluyendo reportes por WhatsApp, Facebook y SMS. La búsqueda autónoma incluye inspecciones de viviendas y visitas realizadas con la aplicación Vectorpoint, así como reportes a puestos de salud con o sin presencia de insectos. Las formas convencionales incluyen publicaciones en Facebook a toda la ciudad y la búsqueda de viviendas asignadas a través de la aplicación de control. Los reportes a los puestos de salud solo se permiten con presencia de insectos.

El esfuerzo de trabajo de los especialistas y del sistema varió entre los dos grupos, principalmente debido a la activación del sistema de respuesta en el brazo inmune, tras la detección de viviendas infestadas. En el brazo inmune, los especialistas dedicaron 835,2 días-persona a visitar las viviendas guiados por la aplicación Vectorpoint. Se emplearon 202 días-persona adicionales en la respuesta adaptativa alrededor de las 10 infestaciones índice y en la aplicación de insecticidas a las 23 viviendas infestadas y a sus vecinos inmediatos, lo que requirió 48 días-persona, para un total de 1085,2 días-persona (Tabla suplementaria 1).

En el brazo control, los especialistas dedicaron 855,2 días-persona a inspeccionar viviendas previamente asignadas a través de la aplicación de control. No se detectaron viviendas infestadas mediante la búsqueda activa. Se necesitó un día adicional para inspeccionar a los vecinos de la única vivienda índice en este brazo, y se emplearon 8 días-persona en la aplicación de insecticidas a las viviendas infestadas y sus vecinos inmediatos, haciendo un total de 864,2 días-persona de esfuerzo.

La tasa de detección de infestaciones fue significativamente mayor en el grupo inmune (23/1085,2 días-persona) que en el grupo de control (5/864,2 días-persona); (Razón de tasas = 3,66 [IC del 95 %: 1,35 - 12,38], valor p = 0,0062, prueba exacta de Fisher).

**Table 2.** Características iniciales de los grupos de vigilancia de cada brazo, medidas durante la fase de ataque de la campaña de control de vectores de la enfermedad de Chagas en Arequipa, Perú (en blanco). Resultados provisionales del tercer año de un estudio comparativo entre los sistemas de vigilancia “Inmune” y “Convencional” (en gris).

| **Pareja balanceada de Cluster** | **Inmune** | | | | | | **Convencional** | | | | | |
| --- | --- | --- | --- | --- | --- | --- | --- | --- | --- | --- | --- | --- |
|  | **Año de la fase de ataque** | **Positivas durante la fase de ataque** | **Viviendas tratadas** | **Viviendas totales** | **Foci detectados** | **# de viviendas infestadas** | **Año de la fase de ataque** | **Positivas durante la fase de ataque** | **Viviendas tratadas** | **Viviendas totales** | **Foci detectados** | **# de viviendas infestadas** |
| 1 | 2012 | 115 | 2626 | 3265 | 1 | 2 | 2012 | 83 | 1935 | 2901 | 0 | 0 |
| 2 | 2011 | 72 | 1254 | 1635 | 0 | 0 | 2011 | 68 | 1113 | 1579 | 0 | 0 |
| 3 | 2007 | 89 | 865 | 1874 | 0 | 0 | 2009 | 115 | 1015 | 2106 | 0 | 0 |
| 4 | 2007 | 31 | 316 | 1577 | 0 | 0 | 2007 | 26 | 844 | 1377 | 0 | 0 |
| 5 | 2011 | 142 | 1304 | 1760 | 0 | 0 | 2012 | 160 | 1061 | 1376 | 0 | 0 |
| 6 | 2012 | 50 | 727 | 2760 | 0 | 0 | 2012 | 102 | 2108 | 2718 | 0 | 0 |
| 7 | 2009 | 6 | 398 | 2482 | 0 | 0 | 2009 | 49 | 925 | 2483 | 0 | 0 |
| 8 | 2007 | 304 | 1551 | 2231 | 0 | 0 | 2007 | 423 | 1615 | 1655 | 0 | 0 |
| 9 | 2011 | 430 | 3204 | 4716 | 1 | 1 | 2007 | 193 | 1313 | 3987 | 0 | 0 |
| 10 | 2007 | 14 | 152 | 1247 | 0 | 0 | 2007 | 27 | 742 | 1032 | 0 | 0 |
| 11 | 2015 | 33 | 969 | 1484 | 0 | 0 | 2012 | 0 | 0 | 1188 | 0 | 0 |
| 12 | 2009 | 54 | 1082 | 1280 | 0 | 0 | 2009 | 56 | 607 | 1215 | 0 | 0 |
| 13 | 2009 | 287 | 1768 | 2056 | 1 | 3 | 2009 | 264 | 1657 | 1968 | 0 | 0 |
| 14 | 2012 | 16 | 553 | 2492 | 0 | 0 | 2012 | 6 | 117 | 2583 | 0 | 0 |
| 15 | 2005 | 196 | 2872 | 3291 | 1 | 4 | 2006 | 249 | 2083 | 3095 | 0 | 0 |
| 16 | 2014 | 6 | 184 | 1874 | 0 | 0 | 2014 | 50 | 1502 | 2106 | 0 | 0 |
| 17 | 2005 | 58 | 1010 | 1023 | 1 | 1 | 2007 | 105 | 1084 | 1109 | 0 | 0 |
| 18 | 2012 | 108 | 2265 | 4194 | 2 | 8 | 2014 | 42 | 2217 | 4377 | 1 | 5 |
| 19 | 2007 | 117 | 1199 | 1233 | 0 | 0 | 2007 | 102 | 859 | 1181 | 0 | 0 |
| 20 | 2005 | 249 | 2606 | 2639 | 0 | 0 | 2007 | 175 | 1017 | 2405 | 0 | 0 |
| 21 | 2007 | 92 | 1399 | 2673 | 0 | 0 | 2007 | 61 | 1629 | 2860 | 0 | 0 |
| 22 | 2015 | 12 | 1088 | 5334 | 0 | 0 | 2015 | 69 | 1483 | 6734 | 0 | 0 |
| 23 | 2009 | 57 | 1033 | 3121 | 1 | 1 | 2012 | 0 | 26 | 3871 | 0 | 0 |
| 24 | 2014 | 141 | 3025 | 3529 | 1 | 1 | 2015 | 66 | 1761 | 3642 | 0 | 0 |
| 25 | 2015 | 5 | 225 | 1760 | 0 | 0 | 2015 | 21 | 579 | 1740 | 0 | 0 |
| 26 | 2007 | 139 | 1316 | 1491 | 1 | 2 | 2007 | 126 | 1443 | 1560 | 0 | 0 |
| 27 | 2012 | 71 | 1441 | 1699 | 0 | 0 | 2012 | 87 | 1516 | 1777 | 0 | 0 |
| 28 | 2006 | 22 | 649 | 2098 | 0 | 0 | 2005 | 39 | 653 | 1658 | 0 | 0 |
| 29 | 2005 | 37 | 838 | 983 | 0 | 0 | 2005 | 37 | 977 | 983 | 0 | 0 |
| 30 | 2005 | 3 | 92 | 471 | 0 | 0 | 2009 | 36 | 432 | 735 | 0 | 0 |
| **Mean** | | **98.5** | **1267** | **2275.7** |  |  |  | **94.6** | **1143.8** | **2266.7** |  |  |
| **TOTAL** | | **2956** | **38011** | **68272** | **10** | **23** |  | **2837** | **34313** | **68001** | **1** | **5** |

**Discusión**

Cuestionamos formalmente el paradigma convencional de control de vectores, comparándolo con un enfoque alternativo inspirado en el sistema inmunitario adaptativo, y la nueva intervención superó con creces a la anterior. Incluso cuando se reforzaron con una docena de años de datos previos, potencia computacional y modelos espaciales de los que rara vez disponen las agencias de control de vectores, y con el mismo personal experimentado que el grupo de control inmunitario, los protocolos convencionales solo lograron detectar un único foco de infestación de vectores en cinco viviendas afectadas. Durante el mismo periodo, el equipo inmunológico detectó 23 hogares infestados en 10 focos, distribuidos en nueve sectores de la ciudad.

El control de vectores urbanos requiere vigilancia y control a diversas escalas, ya que los insectos se propagan entre viviendas, manzanas, barrios y ciudades. El sueño del control convencional ha sido durante mucho tiempo la escalabilidad: la capacidad de establecer un conjunto de protocolos que, en palabras de Anna Tsing, “puedan expandirse —y expandirse, y expandirse— sin replantearse los elementos básicos” [32].  Este tipo de planteamientos es habitual en la ampliación de proyectos piloto a programas de gran envergadura [33], en la planificación inicial de la Iniciativa del Cono Sur para eliminar *Triatoma infestans*, y en el énfasis puesto en la normalización de las estrategias entre los Estados miembros [34].

El sistema inmunitario siempre actúa a diferentes escalas. En general, el mismo sistema que protege a un ratón también protege a un alce, pero no es escalable: no se puede trasladar sin esfuerzo de un lugar a otro, ni expandirse a territorios cada vez más amplios. Nuestros protocolos en el brazo inmune permitieron a los técnicos tomar decisiones basadas en información que nunca encajaría en un modelo estadístico. Esa libertad es fundamental: a medida que se avanza hacia la eliminación de los insectos, se detectan y se eliminan las infestaciones predecibles, dejando atrás las idiosincrásicas. El brazo inmune también se basó en gran medida en la comunicación y las relaciones personales —ya fuera a través de AlertaChirimacha o de la comunicación puerta a puerta—, lo cual no se pueden escalarse fácilmente. La necesidad de este tipo de adaptación en la vigilancia y el control de vectores es cada vez más reconocida en el control de la enfermedad de Chagas [34], y, en términos más generales, los llamamientos a la normalización han sido sustituidos por llamamientos a prestar mayor atención a las ecologías, geografías y políticas locales [36].

Las descripciones del propio sistema inmunitario están llenas de analogías y metáforas. Los linfocitos T y los patógenos fueron alguna vez descritos como “en guerra” entre sí [37] y, más tarde, se dividieron en “propio” y “ajeno”, como si fueran una nación con una frontera, de la cual un lado necesita protección frente al otro [38]. La analogía propio/ajeno ha caído en desuso con el reconocimiento creciente de la importancia de la microbiota, un conjunto de organismos que no pueden asignarse de manera fácil ni consistente como propios o extraños [39]. La comprensión del sistema inmune, así como las metáforas utilizadas para transmitirla, continuará evolucionando, y las reconceptualizaciones podrían generar nuevas ideas sobre cómo controlar vectores y otros agentes causantes de enfermedad.

Para la vigilancia vectorial de la enfermedad de Chagas, los reportes de la comunidad suelen ser más sensibles que la búsqueda activa realizada por personal capacitado [40,41]. En el brazo inmune, el personal de control de vectores no insistía en ingresar a los hogares, sino que se enfocaba en la comunicación y la difusión de materiales para facilitar los reportes. El enfoque convencional se basa en cuotas: se debe inspeccionar un número determinado de viviendas. Para que el enfoque inmune se adopte formalmente, el sistema de cuotas, que se encuentra dentro de un marco de pago por desempeño [42–44], tendría que ser ampliamente modificado o eliminado. Las cuotas tienen un historial lamentable en Perú: en la década de 1990 se implementaron programas de esterilización basados en cuotas, lo que derivó en abusos generalizados contra las mujeres, ya que el personal de salud realizaba esterilizaciones por la fuerza o sin el consentimiento adecuado mientras intentaba cumplir con las cuotas [43,45–47]. Las cuotas para ingresar e inspeccionar viviendas son menos nefastas, pero aún provocan comportamientos ineficientes e irracionales.

Nuestro estudio tuvo varias limitaciones. Nuestro protocolo originalmente dependía en gran medida de enfoques basados en la comunidad. Los agentes comunitarios de salud debían actuar como las células T CD4+, monitoreando sus barrios y “expandiéndose clonalmente” al capacitar a otros agentes comunitarios de salud tras detectar un vector. Quizá si COVID-19 y los confinamientos relacionados no hubieran impedido la participación de los agentes de salud comunitarios, estos podrían haber desempeñado un papel fundamental en el nuevo sistema.

Trasladamos nuestra estrategia de comunicación y expansión clonal de los agentes comunitarios de salud a las redes sociales. Al hacerlo, introdujimos contaminación adicional en el diseño del estudio, ya que no pudimos dirigir las publicaciones en redes sociales a una escala espacial consistente en torno a una infestación y, por supuesto, no pudimos evitar que las publicaciones se propagaran a áreas asignadas al brazo de control. De hecho, el Ministerio de Salud volvió a publicar nuestros mensajes con regularidad. Aunque no pudimos evitar esta contaminación, es posible que la hayamos reducido al incluir en nuestras publicaciones los nombres de los barrios afectados. Esta contaminación habría sesgado nuestros resultados hacia la nulidad.

El formalismo de un ensayo aleatorizado también se vio afectado durante la pandemia. El análisis interino que presentamos aquí estaba previsto para el final del tercer año del ensayo, y lo hemos realizado según lo preespecificado al finalizar dicho año. Sin embargo, contamos con menos de tres años de datos disponibles.

Hemos presentado aquí resultados prometedores sobre un nuevo sistema de vigilancia, basado en el sistema inmune de los mamíferos, para controlar un solo insecto en una ciudad. Pero el sistema inmunitario no se enfrenta a un patógeno a la vez y las ciudades ciertamente no tratan los agentes infecciosos de manera aislada. El verdadero poder de la analogía podría revelarse al ampliar su aplicación para abordar múltiples amenazas concurrentes. Lograr esto podría requerir la eliminación progresiva de los sistemas convencionales para liberar recursos y permitir una respuesta más adaptativa y flexible. Aún no está claro si un cambio tan importante en el enfoque será aceptable para las agencias de control, pero el potencial es grande.

#

# **References**

1. Alberts B, editor. Molecular biology of the cell. 4th ed. New York: Garland Science; 2002.
2. Ratajczak W, Niedźwiedzka-Rystwej P, Tokarz-Deptuła B, Deptuła W. Immunological memory cells. cejoi. 2018;43: 194–203. doi:10.5114/ceji.2018.77390
3. Willoughby UE. Chapter 6 “Mosquito or Man?”: Imperialism and the Rise of Tropical Medicine, 1878-1912. Yellow fever, race, and ecology in nineteenth-century New Orleans. Baton Rouge: Louisiana State University Press; 2017. pp. 142–166.
4. Cucunubá ZM, Okuwoga O, Basáñez M-G, Nouvellet P. Increased mortality attributed to Chagas disease: a systematic review and meta-analysis. Parasites Vectors. 2016;9: 42. doi:10.1186/s13071-016-1315-x.
5. Bonney KM. Chagas disease in the 21st Century: a public health success or an emerging threat? Parasite. 2014;21: 11. doi:10.1051/parasite/2014012.
6. Dias JCP. Southern Cone Initiative for the elimination of domestic populations of Triatoma infestans and the interruption of transfusion Chagas disease: historical aspects, present situation, and perspectives. Mem Inst Oswaldo Cruz. 2007;102: 11–18. doi:10.1590/S0074-02762007005000092.
7. Schofield CJ, Dias JCP. The Southern Cone Initiative against Chagas Disease. Advances in Parasitology. Elsevier; 1999. pp. 1–27. doi:10.1016/S0065-308X(08)60147-5
8. Lorca M, Schenone H, Contreras M del C, García A, Rojas A, Valdés J. Evaluación de los programas de erradicación de vectores de la enfermedad de Chagas en Chile mediante estudio serológico de niños menores de 10 años. Bol chil parasitol. 1996;51: 80–5.
9. Silveira A, Vinhaes M. Elimination of vector-borne transmission of Chagas disease. Mem Inst Oswaldo Cruz. 1999;94: 405–411. doi:10.1590/S0074-02761999000700080
10. Uruguay declared free of Chagas disease transmission. TDR News. 1998; 6.
11. Pan American Health Organization. Paraguay interrupts vector transmission of Chagas in the home. 2018. Available: <https://www.paho.org/en/news/8-8-2018-paraguay-interrupts-vector-transmission-chagas-home>
12. Levine R, Kinder M, What Works Working Group. Case 12 Controlling Chagas Disease in the Southern Cone of South America. Millions saved: proven successes in global health. Washington, D.C: Center for Global Development; 2004.
13. Levy MZ, Bowman NM, Kawai V, Waller LA, Cornejo Del Carpio JG, Cordova Benzaquen E, et al. Periurban Trypanosoma cruzi–infected Triatoma infestans, Arequipa, Peru. Emerg Infect Dis. 2006;12: 1345–1352. doi:10.3201/eid1209.051662
14. Gutfraind A, Peterson JK, Billig Rose E, Arevalo-Nieto C, Sheen J, Condori-Luna GF, et al. Integrating evidence, models and maps to enhance Chagas disease vector surveillance. Basáñez M-G, editor. PLoS Negl Trop Dis. 2018;12: e0006883. doi:10.1371/journal.pntd.0006883
15. Levy MZ, Small DS, Vilhena DA, Bowman NM, Kawai V, Cornejo Del Carpio JG, et al. Retracing Micro-Epidemics of Chagas Disease Using Epicenter Regression. Meyers LA, editor. PLoS Comput Biol. 2011;7: e1002146. doi:10.1371/journal.pcbi.1002146
16. Recommendations of the International Task Force for Disease Eradication. MMWR Recomm Rep. 1993;42: 1–38.
17. World Health Organization. Handbook for integrated vector management. 2012; 67.
18. Hong AE, Barbu CM, Small DS, Levy MZ, The Chagas Disease Working Group in Arequipa. Mapping the Spatial Distribution of a Disease-Transmitting Insect in the Presence of Surveillance Error and Missing Data. Journal of the Royal Statistical Society Series A: Statistics in Society. 2015;178: 641–658. doi:10.1111/rssa.12077
19. Evaluacion Programa de Chagas, Chile, 2003. Santigo de Chile; 2003. pp. 74–80.
20. Delgado S, Castillo Neyra R, Quispe Machaca VR, Ancca Juárez J, Chou Chu L, Verastegui MR, et al. A History of Chagas Disease Transmission, Control, and Re-Emergence in Peri-Rural La Joya, Peru. Gürtler RE, editor. PLoS Negl Trop Dis. 2011;5: e970. doi:10.1371/journal.pntd.0000970
21. Hunter GC, Borrini-Mayorí K, Ancca Juárez J, Castillo Neyra R, Verastegui MR, Malaga Chavez FS, et al. A Field Trial of Alternative Targeted Screening Strategies for Chagas Disease in Arequipa, Peru. Gürtler RE, editor. PLoS Negl Trop Dis. 2012;6: e1468. doi:10.1371/journal.pntd.0001468
22. Levy MZ, Bowman NM, Kawai V, Plotkin JB, Waller LA, Cabrera L, et al. Spatial Patterns in Discordant Diagnostic Test Results for Chagas Disease: Links to Transmission Hotspots. CLIN INFECT DIS. 2009;48: 1104–1106. doi:10.1086/597464
23. Delgado S, Ernst KC, Pumahuanca MLH, Yool SR, Comrie AC, Sterling CR, et al. A country bug in the city: urban infestation by the Chagas disease vector Triatoma infestans in Arequipa, Peru. Int J Health Geogr. 2013;12: 48. doi:10.1186/1476-072X-12-48
24. Barbu CM, Buttenheim AM, Pumahuanca M-LH, Calderón JEQ, Salazar R, Carrión M, et al. Residual Infestation and Recolonization during Urban *Triatoma infestans* Bug Control Campaign, Peru1. Emerg Infect Dis. 2014;20: 2055–2063. doi:10.3201/eid2012.131820
25. Rose EB, Roy JA, Castillo-Neyra R, Ross ME, Condori-Pino C, Peterson JK, et al. A real-time search strategy for finding urban disease vector infestations. Epidemiologic Methods. 2020;9: 20200001. doi:10.1515/em-2020-0001
26. Arevalo-Nieto C, Sheen J, Condori-Luna GF, Condori-Pino C, Shinnick J, Peterson JK, et al. Incentivizing optimal risk map use for Triatoma infestans surveillance in urban environments. Mutheneni SR, editor. PLOS Glob Public Health. 2022;2: e0000145. doi:10.1371/journal.pgph.0000145
27. Geresa Arequipa realiza vigilancia y control ante presencia de “chirimachas” que pueden transmitir la enfermedad de Chagas. Gob.pe. 5 Aug 2022. Available: <https://www.gob.pe/institucion/minsa/noticias/638020-geresa-arequipa-realiza-vigilancia-y-control-ante-presencia-de-chirimachas-que-pueden-transmitir-la-enfermedad-de-chagas>. Accessed 12 Sept 2024.
28. Tamayo LD, Paz-Soldán VA, Condori Pino CE, Malaga Chavez FS, Levy MZ, Gonçalves R. Barriers to surveillance and control of re-emergence of the Chagas disease vector Triatoma infestans in Arequipa, Peru. Beatty N, editor. PLoS Negl Trop Dis. 2025;19: e0013373. doi:10.1371/journal.pntd.0013373
29. Tamayo LD, Condori-Pino CE, Sanchez Z, Gonçalves R, Málaga Chávez FS, Castillo-Neyra R, et al. An effective internet-based system for surveillance and elimination of triatomine insects: AlertaChirimacha. Villinger J, editor. PLoS Negl Trop Dis. 2023;17: e0011694. doi:10.1371/journal.pntd.0011694
30. Harris TH, Banigan EJ, Christian DA, Konradt C, Tait Wojno ED, Norose K, et al. Generalized Lévy walks and the role of chemokines in migration of effector CD8+ T cells. Nature. 2012;486: 545–548. doi:10.1038/nature11098
31. Lu B, Greevy R, Xu X, Beck C. Optimal Nonbipartite Matching and Its Statistical Applications. The American Statistician. 2011;65: 21–30. doi:10.1198/tast.2011.08294
32. Tsing AL. On Nonscalability: The Living World Is Not Amenable to Precision-Nested Scales. Common Knowledge. 2012;18: 505–524. doi:10.1215/0961754X-1630424
33. Shah S. The fever: how malaria has ruled humankind for 500,000 years. 1st ed. New York: Picador; 2010.
34. Silveira AC, Rojas de Arias A, Segura E, Guillén G, Russomando G, Schenone H, et al. El control de la enfermedad de Chagas en los países del Cono Sur de América. Historia de una iniciativa internaciona. 1991/2001. 2002. Available: <https://www3.paho.org/Spanish/AD/DPC/CD/dch-historia-incosur.PDF>
35. World Health Organization, UNICEF/UNDP/World Bank/WHO Special Programme for Research and Training in Tropical Diseases. Global vector control response 2017-2030. Geneva: World Health Organization; 2017. Available: <https://iris.who.int/handle/10665/259205>
36. Moncayo Á, Silveira AC. Current Trends and Future Prospects for Control of Chagas Disease. American Trypanosomiasis. Elsevier; 2010. pp. 55–82. doi:10.1016/B978-0-12-384876-5.00004-6
37. Turney J. Why we should guard against military notions of immunity. aeon. 28 Mar 2016. Available: <https://aeon.co/essays/why-we-should-guard-against-military-notions-of-immunity>. Accessed 10 Sept 2024.
38. Tauber AI. Immunity: The Evolution of an Idea. Oxford University Press; 2017. doi:10.1093/acprof:oso/9780190651244.001.0001
39. Kriegel MA. Self or non-self? The multifaceted role of the microbiota in immune-mediated diseases. Clinical Immunology. 2015;159: 119–121. doi:10.1016/j.clim.2015.05.010
40. Silva RA, Bonifácio PR, Wanderley DMV. Doença de Chagas no Estado de São Paulo: comparação entre pesquisa ativa de triatomíneos em domicílios e notificação de sua presença pela população em área sob vigilância entomológica. Rev Soc Bras Med Trop. 1999;32: 653–659. doi:10.1590/S0037-86821999000600007
41. Gürtler RE, Cecere MC, Canale DM, Castañera MB, Chuit R, Cohen JE. Monitoring house reinfestation by vectors of Chagas disease: a comparative trial of detection methods during a four-year follow-up. Acta Tropica. 1999;72: 213–234. doi:10.1016/S0001-706X(98)00096-5
42. Oficina General de planeamiento presupuesto y modernización, Ministerio Nacional de salud del Peru. Programa Presupuestal 0017: Enfermedades Metaxénicas y Zoonosis Reporte de seguimiento anual 2022. 2022. Available: <http://www.minsa.gob.pe/presupuestales/doc2022/reporte-seguimiento/Reporte%20al%20I%20Semestre%202022_PP_0017.pdf>
43. Ewig C. Second-wave neoliberalism: gender, race, and health sector reform in Peru. University Park, Penn: Pennsylvania State University Press; 2010.
44. Ministerio de Salud del Peru. Anexo 2 Programa Presupuestal 0017: Enfermedades Metaxenicas y Zoonosis. 2016. Available: <https://www.minsa.gob.pe/presupuestales/doc2021/ANEXO2_4.pdf>
45. Cevasco G, Comité de América Latina y el Caribe para la Defensa de los Derechos Humanos de la Mujer, editors. Nada personal: reporte de derechos humanos sobre la aplicación de la anticoncepción quirúrgica en el Perú, 1996 - 1998. Lima; 1999.
46. Urrunaga JM. Siguen obligando a captar pacientes para ligaduras. El comercio. 4 Mar 1998. Available: <https://1996pnsrpf2000.wordpress.com/wp-content/uploads/2012/10/el-comercio-4-de-marzo-de-1998.pdf>. Accessed 8 Sept 2024.
47. Nada personal. Implementación de la Anticoncepción Quirúrgica en el Perú. 1999. Available: <https://www.youtube.com/watch?v=QRcU_JZgatw>
