# Supplementary material for: An immune system for the city: A cluster-randomized trial of a new paradigm for surveillance and control of disease vectors
Source: PLoS Negl Trop Dis. 2026 Jun 22;20(6):e0014464. doi: 10.1371/journal.pntd.0014464 (PMC13340813; doi:10.1371/journal.pntd.0014464)
Supplement: Attachment — Submitted filename: Response to Reviewers (1).pdf [file pntd.0014464.s004.pdf]

May 13th, 2026

Editorial Staff
PLOS Neglected Tropical Diseases

Dear Editors,

Thank you for your consideration of our manuscript *"An Immune System for the City: A Cluster Randomized Trial of a New Paradigm for Surveillance and Control of Disease Vectors"* (PNTD-D-26-00072). We respond to the reviewers' comments and suggestions below in blue, along with the relevant portions of the revised text.

We thank the reviewers for their thorough reading and helpful critique of our work.

Yours sincerely,

Michael Z. Levy

Professor
Department of Biostatistics & Epidemiology
University of Pennsylvania, SOM
714 Blockley Hall, 423 Guardian Drive
Philadelphia, PA 19104-6021
(215) 746-8131 (office)
mzlevy@pennmedicine.upenn.edu

Reviewer #1: Major comments

1. I'd recommend editing the figure (which is central to understanding the design) to make the surveillance components (CHWs, cell phone alert system, vector control technician) more prominent, e.g. bolded larger font, compared to the immune system analogues (antigen presenting cells, CD4+ Th cells, etc). This would be in line with panel A and would highlight the real world components.

Thank you for this suggestion. We revised Figure 1 to give greater prominence to the surveillance system's operational components.

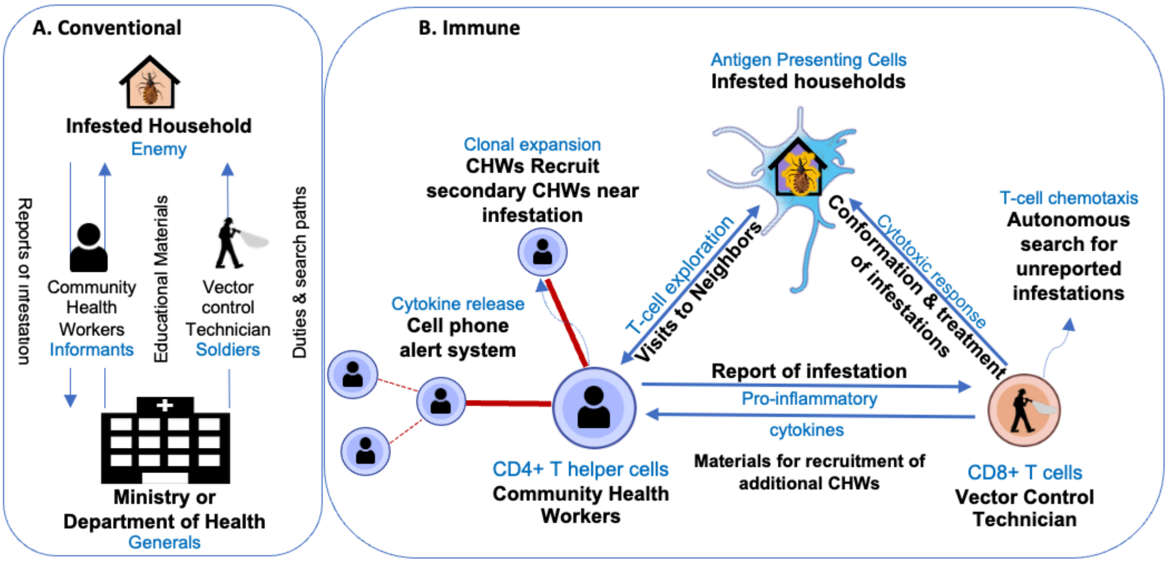

2. If I understand the text correctly, an infested house was defined as a demonstration of vectors either by the householder or the vector tech for the conventional arm but could be defined solely by the householder reporting infestation for the intervention arm. Am I reading that correctly? Was there a component of ground-truthing (that is, some kind of confirmation of vector presence in proportion of reportedly infested houses)? Please clarify these points.

Thank you for your comment. In both the conventional and immune arms, a household was classified as infested based on confirmation of the presence of triatomine vectors by our trained staff. While the reporting pathways differed between arms, the mode of confirmation of infestation was the same.

Both arms (immune and conventional) included components of passive (reports) and active (door to door search) surveillance. But again, no matter the arm or the means by which insects were discovered, the same trained staff confirmed the infestations.

To clarify this, we will add the following to the method section under **Trial Design**:

> *"The primary outcome of the trial was the presence of insects in a household. All instances of insects in households were confirmed by trained study staff in both the*

immune and conventional arms, and regardless of the mode by which the insects were discovered (active or passive surveillance). A secondary outcome was the number of foci of infestation, where all infested households within 200m of each other were considered a focus."

3. In a related doubt, I am not sure I entirely understand the roles played by the vector tech in the intervention arm – did they perform house searches only when asked by householder? How many of those were initiated? It would be useful in the description of the systems to be explicitly list their roles under each arm.

The vector technicians in both arms visited houses proactively (without a report from a homeowner). The difference was in the conventional arm the technician was assigned the exact houses to visit (using our model and an app called PuntosAzules) while in the intervention the technicians were given an app (VectorPoint) that showed risk estimates (from the same model), but allowed to make their own decisions on which houses to visit. We clarified in the revision:

'We used two apps, both based on the same spatio-temporal risk models [25]. The conventional app assigned households for inspection to personnel, providing alternates as needed. The immune app, called VectorPoint, provided risk estimates but allowed inspectors to use their own judgment when searching their areas for insects'

4. Section headed "Response" last 3 lines. Were these facebook pages open to all? Were vector techs told to monitor the page at some interval while activated?

Thank you for this comment. The Facebook page was publicly accessible to all users and was monitored throughout the study by members of the research team (LDT and CECP). The team responded to all inquiries either through public comments, which addressed general questions and provided information, or through private messages, which addressed reports and requests for locations to facilitate the response. To clarify this process, we have revised the Response section of the Methods as follows:

The Facebook page and posts were publicly accessible. LDT and CECP actively monitored the posts and responded to comments or questions. Comments received on the posts were addressed through public replies, while reports of potential infestations were managed through direct communication with users.

5. "Immune suppression during covid-19" You might discuss the adaptability of the novel surveillance system as a potential strength and how to anticipate and maximize its utility.
Yes, some of the immune interventions worked better during the pandemic--but so much happened that we don't want to oversell it.

6. Discussion: I can see clearly how the immune system model of surveillance might be applied to household insect infestations such as bed bugs, but less clearly for other disease vectors, even those where vector population control plays a significant role (tsetse, simulium). Could you comment more specifically on the kinds of surveillance adaptations that might be applicable for other vector-borne diseases?

We agree. We hesitate to make suggestions for systems and places that we don't know--our hope is that our analogy might be helpful to experts controlling these and other vectors to think about control issues a different way, whether by the immune analogy we describe here, or perhaps other analogies that might be more useful.

We have revised the abstract to make it more cautious and to decrease the suggestion that the analogy we used for Chagas vectors in a city would work elsewhere for other vectors without significant tailoring to the complexity of those insects.

> *'Vector surveillance approaches modeled after the immune system are a potentially more effective alternative to conventional approaches, especially to control vector borne diseases in cities and other complex civilian environments.'*

Minor comments

7. Section headed "Intervention design": I had to read this sentence "we adapted aspects of the immune system from the scale of cells to that of landscapes" several time to understand it. Perhaps clearer to say something like "we adapted cell-level components of the immune system to analogues at the scale of landscapes…"

Thank you, we incorporated your suggested rewording.

8. Section headed "Response", 3rd line. If the definition of an infested house could be based solely on householder response, you may want to replace "detection" with "report".

Thank you for this suggestion. In both study arms, households could be identified either through householder reports or through active search conducted by vector control personnel. However, a report alone was not sufficient to classify a household as infested or to trigger a response; all reported infestations were followed up and confirmed by trained staff prior to any intervention. To improve clarity, we revised the wording of the paper. We now use "report" to refer to a householder report and "detection" to refer to confirmed household infestations detected by our vector control personnel. In places we use 'uncovering infestation' as a neutral term including both reports and detections.

9. Figure 2: I realize the Ns are too small to assess this, but I wonder if the large size of 2 of the 3 clusters identified by health post report in both arms together, compared to the cluster sizes detected by the other methods reflects low sensitivity for the health post method (i.e. it generally picks up large clusters, not small ones). Probably too speculative to say anything in this paper, but perhaps when you have more data you could revisit this question.

Thank you for this observation. We also see a signal that health post-based reports are more likely to identify larger infestation clusters. However, as you note, the number of instances in this study is too small to fully assess the hypothesis.

Reviewer #1: This manuscript presents the design and preprogrammed interim evaluation of a surveillance system for household triatomine infestation modeled by analogy on the mammalian immune system. This is, as far as I know, an entirely original way of conceptualizing a surveillance system. While its direct applicability to other disease vectors is likely limited, the creativity of its novel design may well aid in developing novel designs for other vectors, and vector-borne and other diseases in the latter stages of elimination programs.

Yes, thank you. Our aim is to promote rethinking of surveillance in general. The immune analogy worked well for us, but other analogies may be more useful for other vectors and other contexts, and our main hope is to show that new approaches can outperform the conventional system, and, as you say, hope what we did inspires other novel designs.

Reviewer #2: Levy et al. conducted a cluster-randomized field trial to test the efficacy of two interventions during the surveillance phase in the city of Arequipa, Peru: the conventional top-down approach used by vector control programs across Latin America, and an innovative approached patterned after the workings of the immune system. The analogy is nice; the size of the trial and amount of field work daunting, and the preliminary outcomes worthy of being reported.
Thank you! The field work was kind of daunting.

In the last sentence of the Abstract, the authors provided a tentative conclusion: "Vector surveillance approaches modeled after the immune system may be more effective than conventional approaches, especially in cities and other complex civilian environments." However, in the first paragraph of the Discussion conveys a different notion: "… the new intervention quickly outperformed the old." It may be so (I wouldn't be surprised at all), but for an interim analysis (as the authors mention) and an outcome measure based on the overall number of infested houses detected relative to the overall search (detection) effort invested, I would suggest a more cautious stance.

We have made the statement significantly more cautious:

> *"Vector surveillance approaches modeled after the immune system are a potentially more effective alternative to conventional approaches, especially to control vector borne diseases in cities and other complex environments. "*

The trial used a matched-pair design which the (interim) analysis should respect.
The trial was balanced, not one-to-one matched, though we used a matching algorithm to create the balanced arms, which we realize is very confusing.

The interim analysis was prespecified for the simple comparisons we present for balanced arms. We have gone ahead and run a poisson regression for matched count data, with an offset of total homes in each cluster, and a random effect on the matched pairs. We think this serves as a nice robustness check. We have added:

in the methods:

Although not specified for the interim analysis, as a robustness check we ran Poisson regression for matched count data, with an offset of person days in each cluster, and a random effect on the matched pairs.

in the results

In the Poisson regression for matched clusters, the per-household rate by which the immune arm detected confirmed infestations was 2.97 times that of the conventional arm, [CIs 1.21 8.89]

There are guidelines for reporting the outcomes of randomized clinical trials. I was slightly puzzled by the lack of basic information in the paper; for example, when was this trial conducted, and more specifically, what is the duration of the follow-up?

Thank you for the comment. We lost a lot of these details when cutting the text. We added a table with the basic info for quick and easy access. More detail is in the trial registry, which we also cite.

Table 1. Trial at a glance

**Trial component**	**Description**
Study period	Planned: March 2020–February 2025; interim analysis planned for March 2023. Actual: October 2021–March 2023.
Study design	Cluster-randomized trial.
Sample size	Sixty clusters were included, with 30 clusters per arm. The median number of households per cluster was 1,872 in the intervention arm and 1,965 in the control arm.
Randomization	Trial arms were balanced by number of households, time since treatment during the attack phase of the control campaign, and prevalence of infestation detected during the attack phase.

Clusters	Clusters were defined as the geographical jurisdictions of primary care health facilities, according to the local Ministry of Health.
Outcome	Number of households with confirmed presence of *Triatoma infestans*, as verified by study staff.

How was the sample size decided? Cluster size? Power?

The original power calculations are very complex due to a paradox in assessing surveillance and control systems--the better the system is at finding infested houses, initially, the fewer new infestations are created to be detected in the future. Below is the relevant text from the original proposal on this issue. As we are presenting the interim analysis, and stopped the trial before the paradox became an issue, we avoided a lot of the complexities.

> "'A naïve evaluation of an intervention [to improve surveillance and control] leads to perverse interpretations because a better surveillance system might uncover and eliminate more cases, while a poorer one would leave cases undetected for a longer period, thereby allowing the creation, and eventual discovery, of more cases. To avoid this paradox, our approach is to estimate the rate of detection and elimination of infestations...' We estimate power via simulation...We simulated expected counts of infestations in each catchment, assuming our hypothesis were true (higher counts in years 1 & 2 in the immune arm, and lower thereafter, and the converse in the conventional arm). We set the mean expectation to 1 instance per catchment per year (low) and 3 instances per catchment per year (high). We set the dispersion parameter of the negative binomial such that 58% of catchments would have no instances of infestation in the lower count, drawing from our historical data. We then analyzed these count data using the negative binomial regression including three covariates (trial arm, year of the trial, and the interaction of the two). To estimate power, we tally the number of 1000 simulations in which the p-value for each covariate is <0.05. Given these assumptions, a 4-year trial with a sample of 32 catchments (16/arm) would provide us with 90.6% power to detect a significant effect of the arm of the trial, and, importantly, 88.8% power to detect a significant effect of both trial arm and the interaction between trial arm and year."

When the trial was delayed, and the sponsor (NIH-NIAID) would not permit us to extend the time period, we added additional clusters, using all those that were available, in hopes of making up for the lost time.

After reading the section on infestation assessment a couple of times, it is not clear to me who and how were the infestations detected in the "immune" arm; were homeowner reports with no need to hand in a triatomine sufficient to declare a house infested?

Only households in which our staff confirmed the presence of insects were counted as infested in the outcome. The same rule was used for both arms of the trial. We have added the following text to clarify, as well as placed the outcome definition in the new table.

> "*The primary outcome of the trial was the presence of insects in a household. All instances of insects in households were confirmed by trained study staff in both the*

immune and conventional arms, and regardless of the mode by which the insects were discovered (active or passive surveillance)."

There is also mention of "inspectors" playing a role in the "immune" arm, but it is not clear to me whether they were meant to corroborate infestations or they just searched for triatomines wherever they felt it was justified.

The inspectors in the immune arm (as well as the conventional) did both--they searched houses proactively, guided by the risk estimates of the app, as well as responded to household reports and confirmed infestations.

In the conventional arm, did the inspectors search all the houses at least once over the study? No, there were far too many houses to visit all of them. We assigned them to visit 10% of households, after consultation with the Ministry of Health. These households were presented to them through the conventional app, if no one was home, an alternative house was presented. We balanced the search effort (number of days in the field) in the immune arm. We have added a supplemental table with the date range and number of days of active search in each cluster. We note, though, that the clusters were 'in' the trial the whole time, as the passive surveillance components were always on.

Specific comments
Title
Should include "…cluster-randomized trial"

Thank you. We have change the title to:

An Immune System for the City: A Cluster-Randomized Trial of a New Paradigm for Surveillance and Control of Disease Vectors

Abstract
Lacks standard information for cluster-randomized trial, such as the number of clusters, the average number of households included in each arm, and the average duration of follow-up. We included some of the info in the revised abstract and have added all these data into the above table with the information as well.

... Clusters consisted of pre-defined geographic jurisdictions of health facilities, and averaged around 1,900 households. Thirty clusters were assigned to the immune arm, and thirty to the conventional arm, balancing on antecedents related to the probability of vector infestation. Following delays, the trial initiated in October 2021. We report here early results from a pre-planned interim analysis scheduled for March 2023. In the intervention (immune) arm 23 infested households were discovered and confirmed in 10 separate foci; in the control arm only 5 infested households were discovered and confirmed, and all were from the same focus. The immune approach was adaptive, and more effort was expended upon detection of an infestation (1085.2 person days in the immune arm vs 864.2 in the conventional; Rate ratio 23/1085.2:5/864.2 = 3.66 [1.35 - 12.38], p-value =0.0062). Vector surveillance

approaches modeled after the immune system are a potentially more effective alternative to conventional approaches, especially to control vector borne diseases in cities and other complex civilian environments.

Introduction

Looks too short. Why not include the information on the study system (the three paragraphs) in the Introduction?

Thank you, that makes sense. We moved the three paragraphs from the study system to the intro.

Can you provide estimates for the prevalence of human infection with T. cruzi and house infestation rates at different time points?

While we don't have systematic city-wide serology, we have added some estimates from studies we conducted during the initial control campaign. We haven't had an infected insect since ~2017, so all the serological cases would have been infected prior to the interventions, and almost certainly prior to the attack phase of the initial campaign. The new text in the intro reads:

Serological studies conducted contemporaneously with the attack phase revealed a range of infection prevalence from 7.6% in a peri-rural district (La Joya) [20], to ~5% in peri-urban districts [13], 2.3% in more central districts (multiple districts) [21] and 1.4% (Mariano Melgar) [22].

Methods
Intervention design.
As both arms include an intervention, following your terminology, using “immune” and “classic” or “vertical” or “conventional” throughout would be clearer than intervention and control arms. Were all actions performed concurrently in both arms? What is the timeline of the onset of interventions and other study components?
In the revised manuscript we use only 'immune' and 'conventional' for the arms. The arms entered the trial concurrently. The active search components took a lot of time, of course, and were rolled out over time, but always balancing the effort of our staff between the two arms. We have included the time periods in the table in the supplement.

How did you establish the number of clusters in each study arm?
The clusters were already established by the Ministry of health and followed political divisions of the city. We initially were going to use a smaller number of these, but following the delay increased the size of the study to include all the clusters that had been in the original attack phase of the control campaign.

We have clarified in the revised abstract and the new table:

> *'Clusters consisted of pre-defined geographic jurisdictions of health facilities, and averaged 2,271 households'*

Under Reporting, the conventional system required that homeowners produced some proof of infestation whereas the immune system did not. This is important and it seems to have been played down throughout the text.
For the purpose of the trial all infestations were confirmed by our study personnel. We agree that reporting was made easier in the immune arm--but this was the idea of the intervention.

Under Search, how were the inspections performed? Search effort, number of people, supervision? This is key to outcome assessment.

We include a new supplemental table with the search effort by arm in each cluster. The search as part of the interventions differ. All inspections start with a discussion at the door with the homeowner. In the control arm the technicians are **required to enter the house** and search all rooms and peri-domestic structures. In the immune arm the technician offers to enter and search the premises, but does not have a quota. We do not use a strict person hour time limit, because the houses vary immensely by size. We clarified at the end of the search section:

> *'In both arms we did not use a strict person hour time limit, because the houses in the study area vary immensely by size.'*

Search for confirmation of infestation was the same under both arms, and was performed by our trained staff and was followed by insecticide application by one technician.

Under Interaction with homeowners, you stress again the suspicion side of the story, probably an excessive emphasis, perhaps you have evidence on this respect, a reference?

Thank you--we have added the missing references to our recent paper in the journal:

Barriers to surveillance and control of re-emergence of the Chagas disease vector Triatoma infestans in Arequipa, Peru and in an earlier paper:

To spray or not to spray? Understanding participation in an indoor residual spray campaign in Arequipa, Peru https://pmc.ncbi.nlm.nih.gov/articles/PMC5491364/

Home searches for triatomine bugs require an interaction with homeowners and consent, hence they are not independent processes.

Agreed--it's a very complex interaction. We note the consent is not for participation in our trial--it is a verbal consent for control personnel to perform routine surveillance and insecticide application. We used the same staff in both arms.

Under Response, “vector technicians were recruited from nearby areas”. Are these people as qualified as vector control personnel or are they neighbors willing to help? Perhaps “technicians” is misleading depending on experience, training support etc. You also use “original technicians”: are these employees of health services? A few sentences below you mention “inspectors”; better be consistent and explain the affiliations of each of these actors.
Sorry this was poorly worded! We didn't recruit new technicians, we just brought our technicians who were working nearby to the site of infestation (the way the immune system recruits cells to the site of infection). We have reworded in the revision:

> *“Vector technicians working in nearby areas were brought to assist in controlling the focus of infestation, the way immune cells are recruited to the site of an infection."*

Last sentence before “Deviations…” is unfinished.
fixed.

Under Deviations…, you mention community health workers of two types, let’s say primary and secondary CHW. The text is sometimes unclear to which of them you refer to. Were they volunteers? Did they have any training? In reference to the three-year follow-up you mention elsewhere, how long did their effective participation last?
Once the pandemic began the community health worker system in Arequipa completely broke down and we were unable to include any Community Health Workers in the trial. But the idea was that the primary CHWs, who do, supposedly or occasionally, receive training (but not money) from the Ministry of Health, would recruit neighbors to assist them temporarily. We were going to provide the primary CHWs training materials and assist, as needed, in their training of the secondary CHWs.

Under Trial design, I suspect “catchment” is equivalent to “cluster”. If so, I’d suggest that you make the equivalence explicit and stick to one term.

Thank you. They are equivalent as the Ministry of Health's service catchments were used to define our study clusters. We ensure we use catchments only when referring to the pre-existing units, or the areas where personnel are assigned for active search, and cluster when speaking about our study design in the revision.

It’s nice that you provide the average number of infested houses in each catchment. Can you provide a measure of precision?

The measure of infestation that we use comes from the data collected during the spray campaign. During this campaign we attempted to treat every house, so it's not data from a

random sample amenable to confidence intervals. Some households did not participate in the treatment of course, and the non-participants are not the same as the participants.

Results

Figure 2 caption. Revise the wording of the last sentence.

We have revised the caption:

> **Figure 2.** Infestations detected, by modality of discovery of the index household, under the immune and conventional surveillance arms. *Immune modalities*: AlertaChirimacha includes reports via Whatsapp, Facebook & text. Autonomous search includes inspections of households and visits conducted with the Vectorpoint application. Reports to health posts, with or without an insect. *Conventional Modalities* include Facebook posts to the whole city, Search of households assigned through the conventional app, and reports to the Health posts with an insect.

Paragraph immediately before Table 1 captions: no homeowner report of infestation?

Thank you for catching that error. We added

> *'One infested household was reported to the health post.'*

Last paragraph of Results: please check the use of Fisher's exact test for comparison of two rate ratios, this may not be right.

We ran a Miettinen-Nurminen test which might be more appropriate for the CIs than Fisher's and include the results in the revision: p-value 0.0038 1.49 - 10.60.

Discussion

First paragraph: why "quickly"? The Results section did not provide data on the performance of both interventions over time.

Thank you. We removed the word 'quickly' and added 'significantly'

Use of "intervention arm" may be slightly misleading. Last sentence conveys essential information on trial outcomes: the 5 infested houses in the conventional arm had triatomines found, whereas the 23 infested houses in the "immune arm" was based on homeowner reports with no delive of a bug?

Sorry again for the confusion. All infestations in both arms, whether by report or otherwise, were confirmed by our study staff in order to be counted in the outcome.

Third paragraph: for those readers not familiar with triatomine bugs, please explain what "idiosyncratic" means here.

The issue isn't related to the bugs--any time control agencies use a model to find and eliminate a problem they find the predictable targets first, and then are left with the peculiar,

or idiosyncratic (as in those that don't follow the usual pattern, that don't have the same 'risk factors').

Limitations first paragraph. Which type of CHW are you referring to? Below you state that they did not play a pivotal role because of covid-19 and the related lockdowns. Can you provide a rough estimate of how long were they involved along the follow-up? This sentence conveys the notion that their participation was marginal, not clear to me.
We had recruited and trained them prior to the epidemic, but once Covid came the whole system broke down. They played no role--0 days-- in the trial reported here. We have clarified in the revision:

> *During the pandemic, the community health worker system broke down and these planned activities were dropped entirely.*

We keep them in only because we think it was a good idea, and hope it gets picked up upon.

At the end of the third paragraph of limitations, you state "But we have less than three years of data on hand." Thank you for being transparent. This issue appeared before. How long is the duration of the follow-up you are reporting? Is it the same for all clusters?

We report here data from October, 2021 through March, 2023. All clusters were in the trial for the whole period. The active search component of both of the systems was staggered--each cluster was searched for a period, proportionate to its size.

Final paragraph is excellent.

Thank you!
