# Supplementary material for: An immune system for the city: A cluster-randomized trial of a new paradigm for surveillance and control of disease vectors
Source: PLoS Negl Trop Dis. 2026 Jun 22;20(6):e0014464. doi: 10.1371/journal.pntd.0014464 (PMC13340813; doi:10.1371/journal.pntd.0014464)
Supplement: Peer review history

# Peer review: Decision Letter 0

**DOI:** 10.1371/journal.pntd.0014464.r001

**Authors:** Audrey Lenhart, Jennifer K. Peterson

25 Mar 2026

PNTD-D-26-00072

An Immune System for the City: A Randomized Trial of a New Paradigm for Surveillance and Control of Disease Vectors

PLOS Neglected Tropical Diseases

Dear Dr. Levy,

Thank you for submitting your manuscript to PLOS Neglected Tropical Diseases. After careful consideration, we feel that it has merit but does not fully meet PLOS Neglected Tropical Diseases's publication criteria as it currently stands. Therefore, we invite you to submit a revised version of the manuscript that addresses the points raised during the review process.

Please submit your revised manuscript by May 24 2026 11:59PM. If you will need more time than this to complete your revisions, please reply to this message or contact the journal office at plosntds@plos.org. When you're ready to submit your revision, log on to https://www.editorialmanager.com/pntd/ and select the 'Submissions Needing Revision' folder to locate your manuscript file.

Please include the following items when submitting your revised manuscript:

* A letter that responds to each point raised by the editor and reviewer(s). You should upload this letter as a separate file labeled 'Response to Reviewers'. This file does not need to include responses to any formatting updates and technical items listed in the 'Journal Requirements' section below.

* A marked-up copy of your manuscript that highlights changes made to the original version. You should upload this as a separate file labeled 'Revised Manuscript with Track Changes'.

* An unmarked version of your revised paper without tracked changes. You should upload this as a separate file labeled 'Manuscript'.

If you would like to make changes to your financial disclosure, competing interests statement, or data availability statement, please make these updates within the submission form at the time of resubmission. Guidelines for resubmitting your figure files are available below the reviewer comments at the end of this letter.

We look forward to receiving your revised manuscript.

Kind regards,

Jennifer K. Peterson

Academic Editor

PLOS Neglected Tropical Diseases

Audrey Lenhart

Section Editor

PLOS Neglected Tropical Diseases

Shaden Kamhawi

co-Editor-in-Chief

PLOS Neglected Tropical Diseases

orcid.org/0000-0003-4304-636XX

Paul Brindley

co-Editor-in-Chief

PLOS Neglected Tropical Diseases

orcid.org/0000-0003-1765-0002

**Journal Requirements:**

We noticed that you used the phrase 'unpublished data' in the manuscript. We do not allow these references, as the PLOS data access policy requires that all data be either published with the manuscript or made available in a publicly accessible database. Please amend the supplementary material to include the referenced data or remove the references.

**Reviewers' comments:**

Reviewer's Responses to Questions

**Key Review Criteria Required for Acceptance?**

As you describe the new analyses required for acceptance, please consider the following:

**Methods:**

-Are the objectives of the study clearly articulated with a clear testable hypothesis stated?

-Is the study design appropriate to address the stated objectives?

-Is the population clearly described and appropriate for the hypothesis being tested?

-Is the sample size sufficient to ensure adequate power to address the hypothesis being tested?

-Were correct statistical analysis used to support conclusions?

-Are there concerns about ethical or regulatory requirements being met?

Reviewer #1: The objectives, study design, population, sample size and analyses are all adequately described with the exception of a few comments I've included below. There are no ethical or regulatory concerns.

Reviewer #2: See Summary below.

**********

**Results**

-Does the analysis presented match the analysis plan?

-Are the results clearly and completely presented?

-Are the figures (Tables, Images) of sufficient quality for clarity?

Reviewer #1: Analysis and results are clear, match the analysis plan with an unavoidable exception which is thoroughly described. I have a suggestion for improving one Figure, outlined below.

Reviewer #2: See Summary below.

**********

**Conclusions**

-Are the conclusions supported by the data presented?

-Are the limitations of analysis clearly described?

-Do the authors discuss how these data can be helpful to advance our understanding of the topic under study?

-Is public health relevance addressed?

Reviewer #1: the conclusions are supported and the discussion is excellent. Public health relevance is addressed.

Reviewer #2: See Summary below.

**********

**Editorial and Data Presentation Modifications?**

Use this section for editorial suggestions as well as relatively minor modifications of existing data that would enhance clarity. If the only modifications needed are minor and/or editorial, you may wish to recommend “Minor Revision” or “Accept”.

Reviewer #1: Major comments

1. I’d recommend editing the figure (which is central to understanding the design) to make the surveillance components (CHWs, cell phone alert system, vector control technician) more prominent, e.g. bolded larger font, compared to the immune system analogues (antigen presenting cells, CD4+ Th cells, etc). This would be in line with panel A and would highlight the real world components.

2. If I understand the text correctly, an infested house was defined as demonstration of vectors either by the householder or the vector tech for the conventional arm but could be defined solely by the householder reporting infestation for the intervention arm. Am I reading that correctly? Was there a component of ground-truthing (that is, some kind of confirmation of vector presence in proportion of reportedly infested houses)? Please clarify these points.

3. In a related doubt, I am not sure I entirely understand the roles played by the vector tech in the intervention arm – did they perform house searches only when asked by householder? How many of those were initiated? It would be useful in the description of the systems to be explicitly list their roles under each arm.

4. Section headed “Response” last 3 lines. Were these facebook pages open to all? Were vector techs told to monitor the page at some interval while activated?

5. “Immune suppression during covid-19” You might discuss the adaptability of the novel surveillance system as a potential strength and how to anticipate and maximize its utility.

6. Discussion: I can see clearly how the immune system model of surveillance might be applied to household insect infestations such as bed bugs, but less clearly for other disease vectors, even those where vector population control plays a significant role (tsetse, simulium). Could you comment more specifically on the kinds of surveillance adaptations that might be applicable for other vectorborne diseases?

Minor comments

7. Section headed “Intervention design”: I had to read this sentence “we adapted aspects of the immune system from the scale of cells to that of landscapes” several time to understand it. Perhaps clearer to say something like “we adapted cell-level components of the immune system to analogues at the scale of landscapes…”

8. Section headed “Response”, 3rd line. If the definition of an infested house could be based solely on householder response, you may want to replace “detection” with “report”.

9. Figure 2: I realize the Ns are too small to assess this, but I wonder if the large size of 2 of the 3 clusters identified by health post report in both arms together, compared to the cluster sizes detected by the other methods reflects low sensitivity for the health post method (i.e. it generally picks up large clusters, not small ones). Probably too speculative to say anything in this paper, but perhaps when you have more data you could revisit this question.

Reviewer #2: See Summary below.

**********

**Summary and General Comments**

Use this section to provide overall comments, discuss strengths/weaknesses of the study, novelty, significance, general execution and scholarship. You may also include additional comments for the author, including concerns about dual publication, research ethics, or publication ethics. If requesting major revision, please articulate the new experiments that are needed.

Reviewer #1: This manuscript presents the design and preprogrammed interim evaluation of a surveillance system for household triatomine infestation modeled by analogy on the mammalian immune system. This is, as far as I know, an entirely original way of conceptualizing a surveillance system. While its direct applicability to other disease vectors is likely limited, the creativity of its novel design may well aid in developing novel designs for other vectors, and vector-borne and other diseases in the latter stages of elimination programs.

Reviewer #2: Levy et al. conducted a cluster-randomized field trial to test the efficacy of two interventions during the surveillance phase in the city of Arequipa, Peru: the conventional top-down approach used by vector control programs across Latin America, and an innovative approached patterned after the workings of the immune system. The analogy is nice; the size of the trial and amount of field work daunting, and the preliminary outcomes worthy of being reported.

In the last sentence of the Abstract, the authors provided a tentative conclusion: “Vector surveillance approaches modeled after the immune system may be more effective than conventional approaches, especially in cities and other complex civilian environments.” However, in the first paragraph of the Discussion conveys a different notion: “… the new intervention quickly outperformed the old.” It may be so (I wouldn’t be surprised at all), but for an interim analysis (as the authors mention) and an outcome measure based on the overall number of infested houses detected relative to the overall search (detection) effort invested, I would suggest a more cautious stance. The trial used a matched-pair design which the (interim) analysis should respect.

There are guidelines for reporting the outcomes of randomized clinical trials. I was slightly puzzled by the lack of basic information in the paper; for example, when was this trial conducted, and more specifically, what is the duration of the follow-up? How was the sample size decided? Cluster size? Power? After reading the section on infestation assessment a couple of times, it is not clear to me who and how were the infestations detected in the “immune” arm; were homeowner reports with no need to hand in a triatomine sufficient to declare a house infested? There is also mention of “inspectors” playing a role in the “immune” arm, but it is not clear to me whether they were meant to corroborate infestations or they just searched for triatomines wherever they felt it was justified. In the conventional arm, did the inspectors search all the houses at least once over the study?

Specific comments

Title

Should include “…cluster-randomized trial”

Abstract

Lacks standard information for cluster-randomized trial such as the number of clusters, average number of households included in each arm, average duration of follow-up.

Introduction

Looks too short. Why not include the information on the study system (the three paragraphs) in the Introduction? Can you provide estimates for the prevalence of human infection with T. cruzi and house infestation rates at different time points?

Methods

Intervention design.

As both arms include an intervention, following your terminology, using “immune” and “classic” or “vertical” or “conventional” throughout would be clearer than intervention and control arms. Were all actions performed concurrently in both arms? What is the timeline of the onset of interventions and other study components?

How did you establish the number of clusters in each study arm?

Under Reporting, the conventional system required that homeowners produced some proof of infestation whereas the immune system did not. This is important and it seems to have been played down throughout the text.

Under Search, how were the inspections performed? Search effort, number of people, supervision? This is key to outcome assessment.

Under Interaction with homeowners, you stress again the suspicion side of the story, probably an excessive emphasis, perhaps you have evidence on this respect, a reference?

Home searches for triatomine bugs require an interaction with homeowners and consent, hence they are not independent processes.

Under Response, “vector technicians were recruited from nearby areas”. Are these people as qualified as vector control personnel or are they neighbors willing to help? Perhaps “technicians” is misleading depending on experience, training support etc. You also use “original technicians”: are these employees of health services? A few sentences below you mention “inspectors”; better be consistent and explain the affiliations of each of these actors.

Last sentence before “Deviations…” is unfinished.

Under Deviations…, you mention community health workers of two types, let’s say primary and secondary CHW. The text is sometimes unclear to which of them you refer to. Were they volunteers? Did they have any training? In reference to the three-year follow-up you mention elsewhere, how long did their effective participation last?

Under Trial design, I suspect “catchment” is equivalent to “cluster”. If so, I’d suggest that you make the equivalence explicit and stick to one term.

It’s nice that you provide the average number of infested houses in each catchment. Can you provide a measure of precision?

Results

Figure 2 caption. Revise the wording of the last sentence.

Paragraph immediately before Table 1 captions: no homeowner report of infestation?

Last paragraph of Results: please check the use of Fisher’s exact test for comparison of two rate ratios, this may not be right.

Discussion

First paragraph: why “quickly”? The Results section did not provide data on the performance of both interventions over time. Use of “intervention arm” may be slightly misleading. Last sentence conveys essential information on trial outcomes: the 5 infested houses in the conventional arm had triatomines found, whereas the 23 infested houses in the “immune arm” was based on homeowner reports with no delive of a bug?

Third paragraph: for those readers not familiar with triatomine bugs, please explain what “idiosyncratic” means here.

Limitations first paragraph. Which type of CHW are you referring to? Below you state that they did not play a pivotal role because of covid-19 and the related lockdowns. Can you provide a rough estimate of how long were they involved along the follow-up? This sentence conveys the notion that their participation was marginal, not clear to me.

At the end of the third paragraph of limitations, you state “But we have less than three years of data on hand.” Thank you for being transparent. This issue appeared before. How long is the duration of the follow-up you are reporting? Is it the same for all clusters?

Final paragraph is excellent.

**********

PLOS authors have the option to publish the peer review history of their article (what does this mean?). If published, this will include your full peer review and any attached files.

If you choose “no”, your identity will remain anonymous but your review may still be made public.

**Do you want your identity to be public for this peer review?**  For information about this choice, including consent withdrawal, please see our Privacy Policy.

Reviewer #1: No

Reviewer #2: No

[NOTE: If reviewer comments were submitted as an attachment file, they will be attached to this email and accessible via the submission site. Please log into your account, locate the manuscript record, and check for the action link "View Attachments". If this link does not appear, there are no attachment files.]

**Figure resubmission:**

While revising your submission, we strongly recommend that you use PLOS’s NAAS tool (https://ngplosjournals.pagemajik.ai/artanalysis) to test your figure files. NAAS can convert your figure files to the TIFF file type and meet basic requirements (such as print size, resolution), or provide you with a report on issues that do not meet our requirements and that NAAS cannot fix.

After uploading your figures to PLOS’s NAAS tool - https://ngplosjournals.pagemajik.ai/artanalysis, NAAS will process the files provided and display the results in the "Uploaded Files" section of the page as the processing is complete. If the uploaded figures meet our requirements (or NAAS is able to fix the files to meet our requirements), the figure will be marked as "fixed" above. If NAAS is unable to fix the files, a red "failed" label will appear above. When NAAS has confirmed that the figure files meet our requirements, please download the file via the download option, and include these NAAS processed figure files when submitting your revised manuscript.

**Reproducibility:**

To enhance the reproducibility of your results, we recommend that authors of applicable studies deposit laboratory protocols in protocols.io, where a protocol can be assigned its own identifier (DOI) such that it can be cited independently in the future. Additionally, PLOS ONE offers an option to publish peer-reviewed clinical study protocols. Read more information on sharing protocols at https://plos.org/protocols?utm_medium=editorial-email&utm_source=authorletters&utm_campaign=protocols

# Author comment: Author response to Decision Letter 1

**DOI:** 10.1371/journal.pntd.0014464.r002

20 May 2026

Attachment Submitted filename: Response to Reviewers (1).pdf

# Peer review: Decision Letter 1

**DOI:** 10.1371/journal.pntd.0014464.r003

**Authors:** Audrey Lenhart, Jennifer K. Peterson, Audrey Lenhart, Jennifer K. Peterson

12 Jun 2026

Dear Dr. Levy,

We are pleased to inform you that your manuscript 'An Immune System for the City: A Cluster-Randomized Trial of a New Paradigm for Surveillance and Control of Disease Vectors' has been provisionally accepted for publication in PLOS Neglected Tropical Diseases.

Before your manuscript can be formally accepted you will need to complete some formatting changes, which you will receive in a follow up email. A member of our team will be in touch with a set of requests.

Please note that your manuscript will not be scheduled for publication until you have made the required changes, so a swift response is appreciated.

IMPORTANT: The editorial review process is now complete. PLOS will only permit corrections to spelling, formatting or significant scientific errors from this point onwards. Requests for major changes, or any which affect the scientific understanding of your work, will cause delays to the publication date of your manuscript.

Should you, your institution's press office or the journal office choose to press release your paper, you will automatically be opted out of early publication. We ask that you notify us now if you or your institution is planning to press release the article. All press must be co-ordinated with PLOS.

Thank you again for supporting Open Access publishing; we are looking forward to publishing your work in PLOS Neglected Tropical Diseases.

Best regards,

Jennifer K. Peterson

Academic Editor

PLOS Neglected Tropical Diseases

Audrey Lenhart

Section Editor

PLOS Neglected Tropical Diseases

Shaden Kamhawi

co-Editor-in-Chief

PLOS Neglected Tropical Diseases

orcid.org/0000-0003-4304-636XX

Paul Brindley

co-Editor-in-Chief

PLOS Neglected Tropical Diseases

orcid.org/0000-0003-1765-0002

***********************************************************

p.p1 {margin: 0.0px 0.0px 0.0px 0.0px; line-height: 16.0px; font: 14.0px Arial; color: #323333; -webkit-text-stroke: #323333}span.s1 {font-kerning: none

Reviewer's Responses to Questions

**Key Review Criteria Required for Acceptance?**

As you describe the new analyses required for acceptance, please consider the following:

**Methods**

-Are the objectives of the study clearly articulated with a clear testable hypothesis stated?

-Is the study design appropriate to address the stated objectives?

-Is the population clearly described and appropriate for the hypothesis being tested?

-Is the sample size sufficient to ensure adequate power to address the hypothesis being tested?

-Were correct statistical analysis used to support conclusions?

-Are there concerns about ethical or regulatory requirements being met?

Reviewer #2: All criteria for acceptance were met.

**********

**Results**

-Does the analysis presented match the analysis plan?

-Are the results clearly and completely presented?

-Are the figures (Tables, Images) of sufficient quality for clarity?

Reviewer #2: All criteria for acceptance were met.

**********

**Conclusions**

-Are the conclusions supported by the data presented?

-Are the limitations of analysis clearly described?

-Do the authors discuss how these data can be helpful to advance our understanding of the topic under study?

-Is public health relevance addressed?

Reviewer #2: All criteria for acceptance were met.

**********

**Editorial and Data Presentation Modifications?**

Use this section for editorial suggestions as well as relatively minor modifications of existing data that would enhance clarity. If the only modifications needed are minor and/or editorial, you may wish to recommend “Minor Revision” or “Accept”.

Reviewer #2: All criteria for acceptance were met.

**********

**Summary and General Comments**

Use this section to provide overall comments, discuss strengths/weaknesses of the study, novelty, significance, general execution and scholarship. You may also include additional comments for the author, including concerns about dual publication, research ethics, or publication ethics. If requesting major revision, please articulate the new experiments that are needed.

Reviewer #2: The authors' revisions are completely satisfactory and the manuscript appears in very good shape for publication.

**********

PLOS authors have the option to publish the peer review history of their article (what does this mean?). If published, this will include your full peer review and any attached files.

If you choose “no”, your identity will remain anonymous but your review may still be made public.

**Do you want your identity to be public for this peer review?**  For information about this choice, including consent withdrawal, please see our Privacy Policy.

Reviewer #2: No

# Editor report: Acceptance letter

**DOI:** 10.1371/journal.pntd.0014464.r004

**Authors:** Audrey Lenhart, Jennifer K. Peterson, Audrey Lenhart, Jennifer K. Peterson

Dear Dr. Levy,

We are delighted to inform you that your manuscript, "

An Immune System for the City: A Cluster-Randomized Trial of a New Paradigm for Surveillance and Control of Disease Vectors," has been formally accepted for publication in PLOS Neglected Tropical Diseases.

We have now passed your article onto the PLOS Production Department who will complete the rest of the publication process. All authors will receive a confirmation email upon publication.

The corresponding author will soon be receiving a typeset proof for review, to ensure errors have not been introduced during production. Please review the PDF proof of your manuscript carefully, as this is the last chance to correct any scientific or type-setting errors. Please note that major changes, or those which affect the scientific understanding of the work, will likely cause delays to the publication date of your manuscript. Note: Proofs for Front Matter articles (Editorial, Viewpoint, Symposium, Review, etc...) are generated on a different schedule and may not be made available as quickly.

Soon after your final files are uploaded, the early version of your manuscript will be published online unless you opted out of this process. The date of the early version will be your article's publication date. The final article will be published to the same URL, and all versions of the paper will be accessible to readers.

For Research Articles, you will receive an invoice from PLOS for your publication fee after your manuscript has reached the completed accept phase. If you receive an email requesting payment before acceptance or for any other service, this may be a phishing scheme. Learn how to identify phishing emails and protect your accounts at https://explore.plos.org/phishing.

Thank you again for supporting open-access publishing; we are looking forward to publishing your work in PLOS Neglected Tropical Diseases.

Best regards,

Shaden Kamhawi

co-Editor-in-Chief

PLOS Neglected Tropical Diseases

Paul Brindley

co-Editor-in-Chief

PLOS Neglected Tropical Diseases
